# Testosterone status following short‐term, severe energy deficit is associated with fat‐free mass loss in U.S. Marines

**DOI:** 10.14814/phy2.15461

**Published:** 2022-09-18

**Authors:** Claire E. Berryman, Holly L. McClung, John J. Sepowitz, Erin Gaffney‐Stomberg, Arny A. Ferrando, James P. McClung, Stefan M. Pasiakos

**Affiliations:** ^1^ Military Nutrition Division US Army Research Institute of Environmental Medicine Natick Massachusetts USA; ^2^ Oak Ridge Institute for Science and Education Belcamp Maryland USA; ^3^ Department of Nutrition and Integrative Physiology Florida State University Tallahassee Florida USA; ^4^ Military Performance Division U.S. Army Research Institute of Environmental Medicine Natick Massachusetts USA; ^5^ Department of Geriatrics, The Center for Translational Research in Aging & Longevity Donald W. Reynolds Institute of Aging, University of Arkansas for Medical Sciences Little Rock Arkansas USA

**Keywords:** eugonadal, hypogonadal, lean mass, weight loss

## Abstract

The objective of this study was to determine metabolic and physiological differences between males with low testosterone (LT) versus those with normal testosterone (NT) following a period of severe energy deficit. In this secondary analysis, 68 male US Marines (mean ± *SD*, 24.6 ± 2.4 y) were dichotomized by testosterone concentration (< or ≥ 10.5 nmol/L as determined from a single blood sample collected between 0600–0630 after an 8–10 h overnight fast by automated immunoassay) following 7 days of near complete starvation (~300 kcal consumed/d, ~85% energy deficit) during Survival, Evasion, Resistance, and Escape (SERE) training. Dietary intake was assessed before (PRE) SERE. Body composition (dual‐energy x‐ray absorptiometry and peripheral quantitative computed tomography) and whole‐body protein turnover (^15^N alanine) were assessed before (PRE) and after (POST) SERE. Mean testosterone concentrations decreased PRE (17.5 ± 4.7 nmol/L) to POST (9.8 ± 4.0 nmol/L, *p* < 0.0001). When volunteers were dichotomized by POST testosterone concentrations [NT (*n* = 24) 14.1 ± 3.4 vs. LT (*n* = 44): 7.5 ± 1.8 nmol/L, *p* < 0.0001], PRE BMI, total fat mass, trunk fat mass, and testosterone were greater and the diet quality score and total carbohydrate intake were lower in NT compared to LT (*p* ≤ 0.05). LT lost more fat‐free mass and less fat mass, particularly in the trunk region, compared to NT following SERE (*p*‐interaction≤0.044). Whole‐body protein synthesis, net balance, and flux decreased and whole‐body protein breakdown increased from PRE to POST in both groups (*p*‐time ≤0.025). Following short‐term, severe energy deficit, Marines who exhibited low testosterone had greater fat‐free mass loss than those who maintained normal testosterone concentrations. Altering body composition and dietary strategies prior to physical training that elicits severe energy deficit may provide an opportunity to attenuate post‐training decrements in testosterone and its associated effects (e.g., loss of lean mass, performance declines, fatigue).

## Graphical abstract

Participants who maintained normal testosterone concentrations (≥ 10.5 nmol/L) following 7 d of severe energy deficit resulting from military training, compared to those who experienced low testosterone concentrations (< 10.5 nmol/L), had 1) greater BMI, total fat mass, trunk fat mass, and testosterone concentrations and a lower diet quality score and total carbohydrate intake at baseline, and 2) less fat‐free mass loss and more fat mass loss, particularly in the trunk region, in response to training.

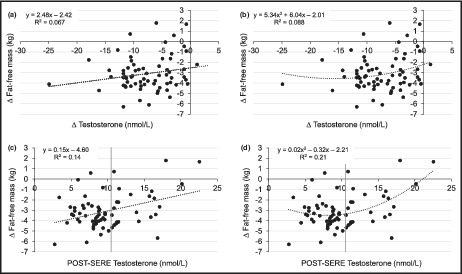

## 1 INTRODUCTION

Testosterone is a sex hormone important for the development of reproductive organs and accretion of muscle and bone mass in males. The harmonized reference range for normal total testosterone concentrations is 10.5–29.5 nmol/L (5th to 95th percentile) in healthy, normal weight males 19–39 y (Travison et al., 2017). Many factors, including physical training and changes in body mass, can influence endogenous testosterone concentrations (Grossmann & Matsumoto, 2017). Prolonged, intense physical training is associated with decreased circulating testosterone due to greater uptake of testosterone by skeletal muscle to promote protein synthesis (via genomic and non‐genomic androgen receptor signaling) and limit stress‐induced protein breakdown (via interference with the glucocorticoid receptor and cortisol binding) (Kraemer et al., 2020). Increased body mass, particularly abdominal fat mass, is associated with low testosterone concentrations (Camacho et al., 2013; He et al., 2018; Nielsen et al., 2007), while weight loss in obese males may improve testosterone (Corona et al., 2013). Conversely, in lean, normal weight individuals, body mass loss results in concurrent decrements in testosterone (Friedl et al., 2000; Henning, Margolis, et al., 2014; Henning, Scofield, et al., 2014; Szivak et al., 2018).

In a previous study, healthy participants subjected to a 21‐day, 40% energy deficit, achieved with increased physical training and decreased food intake, lost 3.2 kg body mass, which coincided with a 16% decrease in total testosterone (Henning, Margolis, et al., 2014; Pasiakos et al., 2013). More extreme energy deficits (> 40%) lasting between 4–62 days, such as those that occur during US Special Operations Forces military training, result in substantial body weight, fat‐free mass, and fat mass loss that coincide with 27%–83% reductions in testosterone (Alemany et al., 2008; Henning, Scofield, et al., 2014; Kyröläinen et al., 2008; Nindl, Alemany, et al., 2007; Nindl, Barnes, et al., 2007; Øfsteng et al., 2020). However, the magnitude of testosterone decrements in response to energy deficit are quite variable, with some males maintaining normal testosterone concentrations and others falling below the normal range (< 10.5 nmol/L) (Alemany et al., 2008). Males who experience low testosterone concentrations in response to energy deficit may have different proportions of fat‐free and fat mass loss than males who maintain normal testosterone concentrations in response to energy deficit, as pharmacologically induced low testosterone concentrations result in fat‐free mass loss and fat mass gain (Thirumalai et al., 2017).

The objective of the current study was to compare metabolic and physiological characteristics of US Marines with low testosterone (defined as below the 5th percentile for normal total testosterone concentrations in healthy, nonobese males 19–39 y, < 10.5 nmol/L) versus those with normal testosterone (≥ 10.5 nmol/L) following Survival, Evasion, Resistance, and Escape (SERE) training. We hypothesized that individuals who maintained normal testosterone concentrations following SERE training would have greater fat mass prior to training and would experience less fat‐free mass loss compared to those with low testosterone.

## 2 METHODS

### 2.1 Experimental overview

This secondary analysis involves data from a larger randomized‐controlled intervention in US Marines participating in SERE training (Berryman et al., 2017). Briefly, demographic and lifestyle questionnaires were administered, body composition was measured, blood samples were obtained, and whole‐body protein turnover was assessed prior to SERE (PRE, baseline). Blood samples were collected and body composition and whole‐body protein turnover measurements were repeated immediately after SERE (POST).

### 2.2 Participant recruitment and enrollment

Individuals ≥18 y participating in U.S. Marine Corps Forces Special Operations Command (MARSOC) SERE school at Camp Lejeune, NC were eligible for the study. Only Marines who successfully completed MARSOC assessment and selection and were assigned to Individual Training Course (ITC) class were eligible for this study. ITC is a physically and mentally challenging 7‐month course designed to produce MARSOC Critical Skills Operators (CSOs), who can operate across the spectrum of special operations in small teams under spartan conditions. ITC is broken down into four training phases. SERE is conducted during Phase 1, which trains and evaluates students in the basic skill sets required of all special operators. The United States Marine Corps is 1 of 8 United States uniformed services (i.e., Marine Corps, Navy, Army, Air Force, Space Force, Coast Guard, Public Health Service Commissioned Corps, and the National Oceanic and Atmospheric Administration Commissioned Officer Corps). Briefly, 125 individuals were briefed, 71 individuals consented, and 68 individuals completed both PRE and POST measures and were included in this secondary data analysis. All volunteers provided written informed consent before participation. This study was approved by the Institutional Review Board at the US Army Research Institute of Environmental Medicine (Natick, MA) and registered at https://clinicaltrials.gov/ as NCT02057094.

### 2.3 SERE school

**FIGURE 1:**
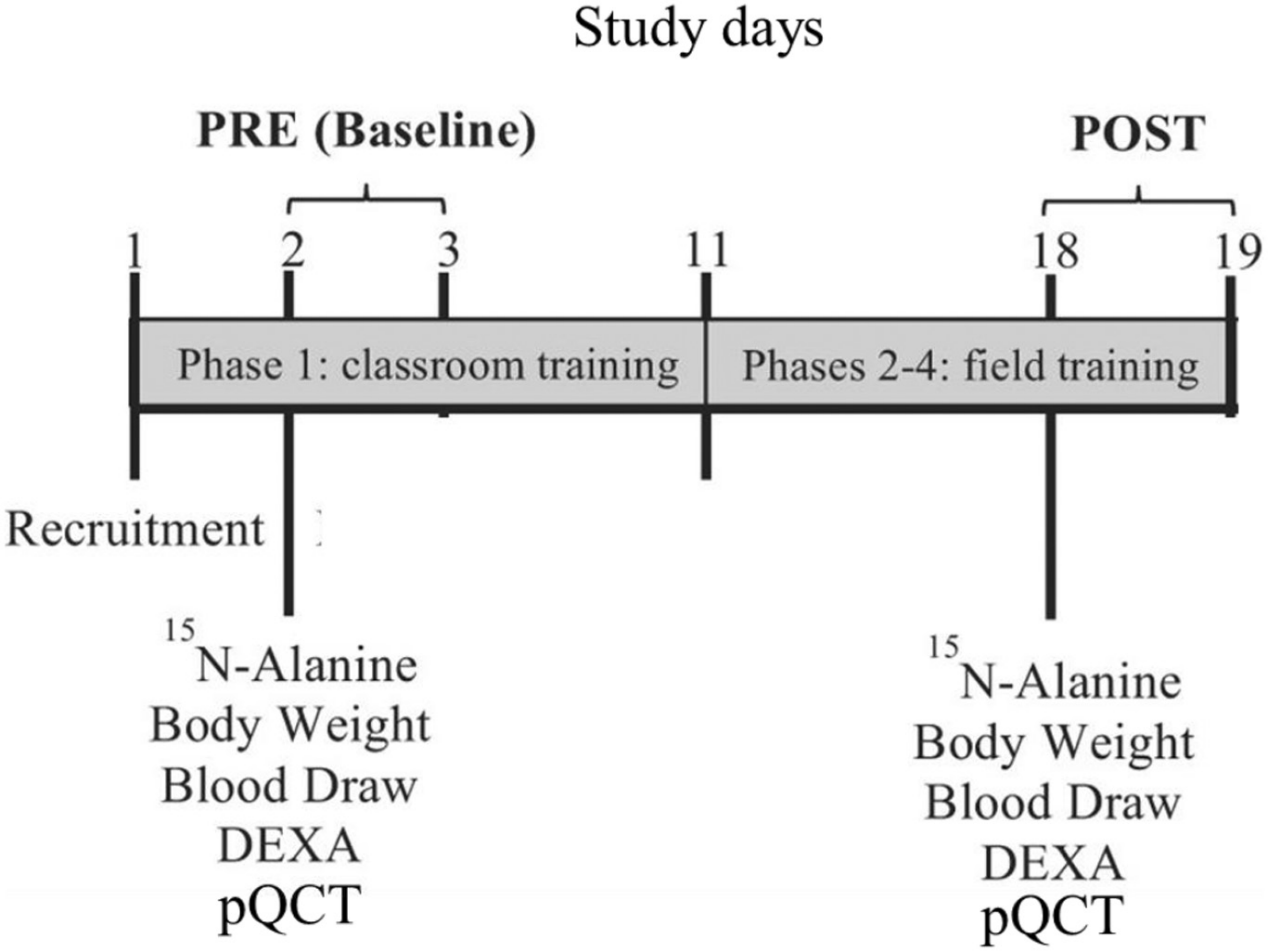
Experimental design. Adapted with permission from (Berryman et al., 2017). DEXA, dual‐energy x‐ray absorptiometry; pQCT, peripheral quantitative computed tomography.

Military personnel at high risk of capture from the enemy (i.e., captivity, isolation, starvation, physical and mental abuse, and exploitation) are required to complete SERE. SERE school consists of 4 phases over ~18 days: (1) 10‐day academic classroom training, (2) 2.5‐day survival skills training, conducted in a natural environment, (3) 2.5‐day evasion training, and (4) 2.5‐days captivity training. Severe energy deficit occurs during phases 2 to 4 (~7 d), which has been described previously (Figure 1) (Berryman et al., 2017). Briefly, participants were provided minimal food during phases 2 and 3 (5 days), which included 1 combat ration (Meal ready‐to‐eat, ~1300 kcal) per participant and a limited amount of vegetables and meat to portion between team members. During phase 2, participants were physically active 14 h/day and, during phase 3, participants were physically active 16 h/day in varied environmental conditions and carrying a pack weighing approximately 20–30 kg. During phase 4, participants were “captured” and placed in a stressful simulated captivity environment. They were provided water on a regular basis but only given two meals during the entirety of phase 4 (~2.5 days); each meal consisted of a piece of bread and approximately one cup of rice. Participants were not physically active during the captivity phase. The intentional limited availability of food (approximately 300 kcal/day) combined with high levels of physical activity (total daily energy expenditure, 4011 ± 475 kcal/d (Sepowitz et al., 2017)) resulted in severe negative energy balance (~85% energy deficit).

### 2.4 Questionnaires

A physical activity and sleep survey [e.g., ‘How often (days/week) in the past 6 months have you normally engaged in aerobic exercise for > 20 min (continuous) at an intensity of > 13 (on a scale from 1 to 20) or 60%–90% of maximal heart rate?’ and ‘How often (days/week) in the past 6 months have you normally engaged in (upper body pushing, upper body pulling, lower body, core) resistance training exercises at an intensity of > 50% of your 1 repetition maximum?’ and ‘During the past 6 months, during the time while you were in garrison only (not deployed), how many hours, on average, of sleep do you get in a 24 hour time period?’] and the Block 2005 food frequency questionnaire (FFQ; NutritionQuest, Berkeley, CA) (Block et al., 1986, 1990) were administered to US Marines at PRE. The Healthy Eating Index‐2010 (HEI) total and 12 component scores [i.e. total fruit, whole fruit, total vegetables, greens and beans, whole grains, dairy, total protein foods, seafood and plant proteins, fatty acids, refined grains, sodium, and solid fats, alcohols, and added sugars (SoFAAS)] were calculated based on the FFQ data as previously described (Guenther et al., 2013; Lutz et al., 2013). For all FFQ variables, participants with implausible energy intakes (< 800 or > 5000 kcal/d) were excluded from those analyses. One participant in the normal testosterone group reported an energy intake >5000 kcal/d at PRE and one participant in the low testosterone group reported an energy intake <800 kcal/d at PRE.

### 2.5 Anthropometric and body composition measures

Height was measured to the nearest 0.1 cm using a stadiometer (Seca; Creative Health Products, Plymouth, MI) at PRE. Body mass was measured to the nearest 0.1 kg using a calibrated digital scale (Befour Model PS6600; Befour, Saukville, WI) at PRE and POST. Basal metabolic rate (BMR) was estimated from height, weight, and age using the Mifflin St. Jeor equation (Mifflin et al., 1990):

In males: 10 × weight (kg) + 6.25 × height (cm) – 5 × age (y) + 5

Total and regional body mass, fat‐free mass, fat mass, and bone mass were measured using dual energy x‐ray absorptiometry (DEXA; Lunar IDXA; GE Lunar, Madison, WI) at PRE and POST. Changes in body energy stores were used to estimate the daily energy deficit (Hoyt et al., 2006):

∆ Energy stores = [∆ fat mass (g) × 9.51 kcal/d + ∆ fat‐free mass (g) × (1 – fat‐free mass hydration) × 4.40 kcal/g] ÷ 7 days,

with fat‐free mass hydration representing the aqueous fraction of fat‐free mass, estimated as 0.73 (Siri, 1956). In a study of male and female military cadets participating in 7 days of strenuous training that led to severe negative energy balance, change in energy stores using the above equation (23.6 ± 3.6 MJ/d; range: 20.1–28.7 MJ/d) did not differ from total daily energy expenditure measured by the doubly‐labeled water technique (23.6 3.4 MJ/d; range: 19.1–27.8 MJ/d) (Hoyt et al., 2006).

Muscle cross‐sectional area (CSA) and intramuscular adipose tissue (IMAT) were measured using peripheral quantitative computed tomography (pQCT; Stratec XCT 3000, Stratec Medizintechnik GmbH, Pforzheim, Germany) of the thigh PRE and POST SERE. Femur length (cm) was measured on the non‐dominant leg using a tape measure from the palpated greater trochanter to the tibial plateau prior to positioning the leg horizontally within the x‐ray gantry. An initial scout view scan was performed using a scan speed of 40 mm/s to identify the distal aspect of the femur and the patella prior to conducting scans at 20% and 50% of the approximated segment length proximal to the distal femoral epiphysis. Slice thickness was 2 mm and voxel size was set at 0.4 mm with a scanning speed of 20 mm/s. Image processing and calculation of muscle CSA and IMAT were performed according to the manufacturer's software package. Prior to analysis, scan images underwent a strong filter (F01F06U01) which combines a 3x3 median filter with a threshold range of −500‐5000 mg × cm^−3^, a 5x5 median filter with a threshold range of −500‐600 mg × cm^−3^ and a 7x7 smoothing filter with a threshold range of −300‐3000 mg x cm^−3^. This filter was chosen based on a prior report indicating strong correlation with CSA measures obtained by MRI (Sherk et al., 2011). Movement artifact in each scan was independently assessed by 3 trained technicians using the visual inspection rating scale previously published by Blew et al. (Blew et al., 2014). Cortical bone area was found using a 710 mg/cm^−3^ threshold, fat was separated from muscle and bone using an outer threshold of −101 mg/cm^−3^, an inner threshold of 40 mg/cm^−3^ and peel mode 2. In order to quantitatively assess movement, bone and positive movement artifact were identified using an outer threshold of 149 mg/cm^−3^ and an inner threshold of 40 mg/cm^−3^, separation mode 4; these parameters allow the inclusion of voxels outside of the periosteal surface that have densities higher than soft tissue, but lower than bone, due to movement. Positive movement artifact was then calculated as the ratio of positive movement area to total bone area. Muscle CSA was calculated as the difference between total area (after removing skin and subcutaneous fat) and bone CSA. All scans for each individual were completed on the same machine. For each volunteer, the reference line for the subsequent scans was placed automatically by the XCT3000 software (Stratec, version 6.2) using the baseline scan image. Calibration of the pQCTs was checked daily using the manufacturer provided cone and cortical phantoms. Test–retest precision of soft tissue measures was determined in our lab by scanning 15 adults on 3 separate occasions with each scan session separated by 1–2 days. The coefficients of variation (CV) for 20% site measures were 2.2% for CSA and 0.8% for muscle density; for the 50% site the CV's were 1.5% for CSA and 0.6% for muscle density.

### 2.6 Sample collection and analysis

A blood sample was collected at both PRE and POST SERE between 0600–0630 and after an 8–10 h overnight fast by antecubital venipuncture. Serum and plasma were isolated, frozen, and shipped on dry ice to the Clinical Chemistry Core at Pennington Biomedical Research Center (Baton Rouge, LA), which is accredited by the Centers for Medicare and Medicaid Services and the College of American Pathologists, operates within the guidelines of Good Clinical Practices, and participates in the CDC Lipid Standardization Program and CDC Accuracy‐Based Monitoring Programs. Blood samples were analyzed for testosterone (reference range for males <50 y: 6–25 nmol/L), sex hormone‐binding globulin (SHBG; male reference range: 10–57 nmol/L), luteinizing hormone (LH; male reference range: 0.8–7.6 IU/L), prolactin (male reference range: 2.5–17.0 μg/L), dehydroepiandrosterone sulfate (DHEAs; male reference range: 2.2–15.2 μmol/L), growth hormone (male reference range: not detectable to 3 μg/L), interleukin‐6 (IL‐6), high‐sensitivity C‐reactive protein (hs‐CRP; reference range: 0.2–11.0 mg/L), insulin (reference range: 42–188 pmol/L), and cortisol (reference range in the morning: 138–690 nmol/L) (Siemens Immulite 2000, Llanberis, UK). Free testosterone (FT) was determined by calculation (Vermeulen et al., 1999). Insulin‐like growth factor 1 (IGF‐1) was analyzed using an enzyme‐linked immunoassay (reference range for 20–29 y: 15–44 nmol/L; ALPCO, Salem, NH). Glucose was analyzed on a Beckman DXC 600 Pro (reference range for ≥19 y: 3.9–6.1 mmol/L; Brea, CA). Neuropeptide Y (NPY) was analyzed using a radioimmunoassay kit (reference range: 30.8–69.3 pmol/L; ALPCO, Salem, NH). Epinephrine (reference range: 0–366 pmol/L) and norepinephrine (reference range: 560–2631 pmol/L) were analyzed using a DLD‐Diagnostika GmbH ELISA kit (Hamburg, Germany). Individual amino acids were measured on an Agilent 1100 Series HPLC (Agilent Technologies, Foster City, CA).

### 2.7 Whole body protein turnover

Whole body protein turnover was measured by a single‐pool whole body method as previously described (Berryman et al., 2017; Ferrando et al., 1996). Briefly, after providing a urine sample to correct for background isotope enrichments, volunteers ingested a single dose of [^15^N]alanine (99% enriched; Cambridge Isotope Laboratories, Andover, MA) at 4 mg ^15^N/kg body mass after consuming their evening meal. Volunteers were instructed to fast and collect their urine for the next 10–12 h, ending with the first void the following morning. Nitrogen flux (Q; g N/24 h) was determined using urinary urea enrichment according to Fern et al. (1985). Protein synthesis (PS) and breakdown (PB) were calculated according to Stein et al. (1991).

### 2.8 Statistical analysis

The current study is a secondary analysis of a randomized‐controlled intervention designed to determine whether an ad libitum diet with supplemental protein, compared with a carbohydrate‐based supplement, would enhance FFM restoration following short‐term severe negative energy balance in US Marines (Berryman et al., 2017). Sample size estimates were based on the primary outcome, FFM restoration (data not shown), and indicated that 19 volunteers/group would provide 90% power to detect between group differences with an effect size of 0.54 and an α of 0.05 (Pasiakos et al., 2013). The current analysis only includes PRE and POST‐SERE testosterone measures, not the 27‐day refeeding and supplementation period, because testosterone concentrations are affected by severe energy deficit but return to normal concentrations with ad libitum feeding (Henning, Scofield, et al., 2014; Nindl et al., 1997). Similarly, in the current study, testosterone concentrations returned to PRE‐SERE levels after the 27‐day refeeding and supplementation period (normal testosterone: 18.5 ± 5.5 and low testosterone: 15.4 ± 4.3 nmol/L).

Normality was assessed for each variable using univariate analysis to quantitatively evaluate skewness and visually inspect box and probability plots. Data was dichotomized based on POST‐SERE testosterone concentrations [< 10.5 nmol/L (low testosterone; *n* = 44) or ≥ 10.5 nmol/L (normal testosterone; *n* = 24)]. Due to a logistical problem during sample collection at PRE, amino acid concentrations could only be measured in a subsample of 29 participants (low testosterone: *n* = 22 and normal testosterone: *n* = 7). Amino acid concentrations were measured in all 68 participants at POST.

A 2‐sided independent t‐test was used to assess differences in baseline characteristics of the testosterone group. Marginal models, with time treated as a repeated measure, were used to assess the effects of testosterone group, time, and their interaction on body composition, blood parameters, and kinetic measures. If a significant interaction effect was observed, post hoc comparisons were adjusted for multiple comparisons using the Bonferroni correction. Pearson and Spearman correlations, linear regression, and 2‐order polynomial regression were used to assess relationships between PRE‐SERE values, POST‐SERE values, and change scores (i.e. POST‐SERE value–PRE‐SERE value). Linear regression analysis was used to assess the ability of Forbes' model [∆FFM/∆TBM = 10.4/ (10.4 + initial fat mass)] to predict change in FFM. Data were analyzed using SAS (Version 9.3; SAS Institute, Cary, NC). Significance was set at *p* < 0.05, and data are presented as means ± *SD*.

## 3 RESULTS

**FIGURE 2:**
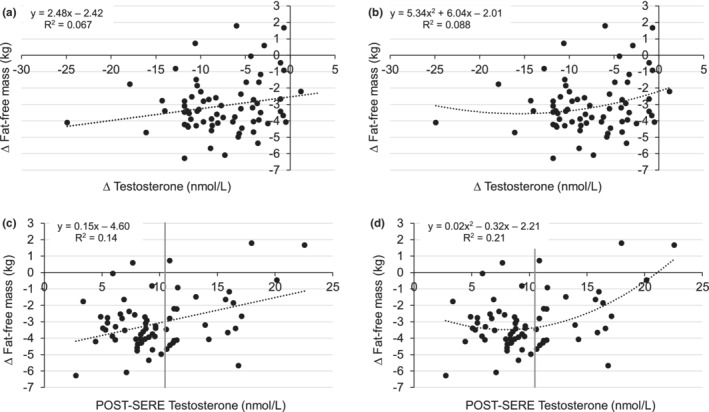
Linear (a) and second‐degree polynomial (b) regression analysis of change in fat‐free mass with change in testosterone concentrations (*R* ^2^ = 0.067, *p* = 0.034 and *R* ^2^ = 0.088, *p* = 0.053, respectively). Non‐transformed data are presented for easier visual interpretation, but the statistics are derived from regression analyses using non‐transformed change in fat‐free mass and log transformed change in testosterone concentrations. Linear (c) and second‐degree polynomial (d) regression analysis of change in fat‐free mass with POST testosterone concentrations (*R* ^2^ = 0.14, *p* = 0.0017 and *R* ^2^ = 0.21, *p* = 0.0005, respectively). The vertical gray line represents the lower end of the harmonized reference range for normal total testosterone concentrations (10.5 nmol/L; 5th percentile) in healthy, normal weight males 19–39 y (Travison et al., 2017).

Volunteers (*n* = 68, mean ± *SD*) were aged 24.6 ± 2.4 y with a total body mass of 83.8 ± 9.3 kg (fat‐free mass: 66.2 ± 6.7 kg; fat mass: 14.1 ± 4.7 kg, 16.6 ± 4.6%) at PRE. Mean testosterone concentrations and fat‐free mass decreased from PRE (17.5 ± 4.7 nmol/L, 66.2 ± 6.7 kg) to POST (9.8 ± 4.0 nmol/L, 63.1 ± 6.5 kg; *p* < 0.0001). Change in fat‐free mass was variable (mean: −3.1 ± 1.7 kg, range: −6.3 to 1.8 kg) and more strongly associated with POST testosterone concentrations than with change in testosterone concentrations (Figure 2a–d; Table S1) or percent change in testosterone concentrations (*R* ^2^ = 0.084, *p* = 0.018). Therefore, participants were dichotomized based on POST testosterone concentrations (< or ≥ 10.5 nmol/L; normal testosterone: 14.1 ± 3.4 vs. low testosterone: 7.5 ± 1.8 nmol/L, *p* < 0.0001). Furthermore, exploratory analyses showed no body composition differences between groups when dichotomized by the median testosterone change (POST‐PRE) or median testosterone percent change (data not shown).

PRE BMI, total fat mass, trunk fat mass, and testosterone were greater and the diet quality score and total carbohydrate intake were lower in the normal testosterone group compared to the low testosterone group (*p* < 0.05; Tables 1 and 2). In addition, prior to SERE, relative protein intake and aerobic exercise tended to be lower in the normal testosterone versus low testosterone group (*p* < 0.06; Table 1).

**TABLE 1:** Pre‐study participant characteristics

	Normal testosterone (≥ 10.5 nmol/L, *n* = 24)	Low testosterone (< 10.5 nmol/L, *n* = 44)
Age, y	25 ± 3	25 ± 2
Weight, kg	85.2 ± 8.3	82.4 ± 9.8
Height, cm	177 ± 6	178 ± 6
BMI, kg/m^2^	27.0 ± 1.6	25.9 ± 2.2*
Basal metabolic rate, kcal/d	1840 ± 114	1817 ± 125
HEI diet quality score	63 ± 11	68 ± 9*
HEI component scores^a^		
Total vegetables	3.6 ± 1.2	4.2 ± 0.9*
Greens and beans	4.0 ± 1.3	4.7 ± 0.7*
Total fruit	3.6 ± 1.4	4.1 ± 1.2
Whole fruit	4.0 ± 1.2	4.2 ± 1.3
Whole grains	3.7 ± 2.2	3.7 ± 2.5
Total dairy	6.7 ± 2.4	6.6 ± 2.2
Total protein	5.0 ± 0.1	4.9 ± 0.3
Seafood/plant protein	4.2 ± 1.0	4.3 ± 1.1
Fatty acids	4.7 ± 2.5	5.5 ± 2.3
Sodium	3.1 ± 2.2	3.4 ± 2.0
Refined grains	9.5 ± 1.0	9.4 ± 1.1
SoFAAS	10.8 ± 4.6	13.2 ± 3.5*
Dietary intake		
Total calories, kcal/d	2304 ± 729	2646 ± 839
Protein, g	105 ± 38	123 ± 43
Protein, g/kg	1.25 ± 0.48	1.51 ± 0.55**
Total fat, g	99 ± 38	109 ± 40
Carbohydrates, g	242 ± 75	294 ± 98*
Exercise habits, d/wk		
Aerobic	4.3 ± 1.2	4.9 ± 1.2**
Upper body pushing	2.8 ± 1.6	3.4 ± 1.6
Upper body pulling	2.4 ± 1.4	2.8 ± 1.6
Lower body	2.1 ± 1.1	2.6 ± 1.6
Core	2.8 ± 1.3	3.5 ± 1.7
Sleep, h/d	6.6 ± 1.1	6.7 ± 0.8

*Note*: Mean ± standard deviation. Independent Student's t‐tests were used to compare means between groups

Abbreviations: HEI, Healthy Eating Index 2010; SoFAAS, solid fats, alcohols, and added sugars.

^a^ Higher scores represent better diet quality. Minimum score for each component is zero. Maximum score for total vegetables, greens and beans, total fruit, whole fruit, total protein, and seafood and plant protein is 5. Maximum score for whole grains, total dairy, fatty acids, sodium, and refined grains is 10. Maximum score for SoFAAS is 20.

* *p* < 0.05.

** *p* < 0.06.

**TABLE 2:** Changes in body composition by post‐SERE hormonal status

	Post‐SERE testosterone status								
	Normal testosterone (*n* = 24, ≥ 10.5 nmol/L)			Low testosterone (*n* = 44, < 10.5 nmol/L)			*p*‐value		
	Pre	Post	Δ	Pre	Post	Δ	Group	Time	Interaction
DEXA measures									
Total body									
Mass, kg	85.4 ± 8.2	79.8 ± 7.7	−5.6 ± 1.0	82.9 ± 9.8	77.0 ± 9.3	−5.8 ± 1.1	0.25	< 0.0001	0.42
Fat‐free mass, kg	65.8 ± 6.5^a^	63.3 ± 6.1^b^	−2.4 ± 2.0	66.4 ± 6.9^a^	62.9 ± 6.9^b^	−3.4 ± 1.3	0.96	< 0.0001	0.016
Fat mass, kg	16.2 ± 4.6^a*^	13.0 ± 4.5^b^	−3.2 ± 1.6	13.0 ± 4.3^a^	10.6 ± 4.2^b^	−2.4 ± 1.2	0.012	< 0.0001	0.021
Bone mass, kg	3.43 ± 0.37	3.43 ± 0.39	0.00 ± 0.07	3.50 ± 0.46	3.50 ± 0.47	−0.00 ± 0.05	0.52	0.92	0.69
Fat mass, %	18.9 ± 4.6	16.2 ± 4.8	−2.7 ± 2.0	15.4 ± 4.1	13.5 ± 4.6	−1.9 ± 1.4	0.008	< 0.0001	0.075
Legs, kg									
Mass	29.4 ± 3.1	28.2 ± 3.1	−1.2 ± 0.9	28.4 ± 4.1	27.1 ± 4.1	−1.3 ± 0.6	0.27	< 0.0001	0.54
Fat‐free mass	22.4 ± 2.4	22.0 ± 2.3	−0.4 ± 0.9	22.4 ± 2.9	21.7 ± 3.0	−0.7 ± 0.7	0.81	< 0.0001	0.13
Fat mass	5.7 ± 1.6	4.9 ± 1.6	−0.8 ± 0.9	4.7 ± 1.6	4.1 ± 1.7	−0.6 ± 0.4	0.027	< 0.0001	0.23
Bone mass	1.30 ± 0.18	1.33 ± 0.16	0.02 ± 0.08	1.35 ± 0.19	1.35 ± 0.20	0.00 ± 0.02	0.51	0.049	0.077
Trunk, kg									
Mass	39.6 ± 3.7	36.0 ± 3.4	−3.5 ± 1.0	38.5 ± 4.5	34.8 ± 4.1	−3.7 ± 1.2	0.28	< 0.0001	0.62
Fat‐free mass	30.2 ± 3.0^a^	28.7 ± 2.9^b^	−1.4 ± 1.4	30.9 ± 3.0^a^	28.8 ± 2.9^b^	−2.1 ± 1.1	0.55	< 0.0001	0.044
Fat mass	8.3 ± 2.5^a*^	6.2 ± 2.2^b^	−2.1 ± 0.9	6.5 ± 2.5^a^	4.9 ± 2.2^b^	−1.6 ± 0.7	0.010	< 0.0001	0.018
Bone mass	1.08 ± 0.14	1.07 ± 0.15	−0.01 ± 0.05	1.09 ± 0.18	1.09 ± 0.19	−0.00 ± 0.05	0.67	0.33	0.64
pQCT measures, cm^2^									
Muscle CSA, 20% site	9407 ± 1227	9124 ± 1176	−282 ± 430	9461 ± 1247	8961 ± 1134	−465 ± 365	0.90	<0.0001	0.068
Muscle CSA, 50% site	19,457 ± 1805	18,536 ± 1571	−1088 ± 599	19,828 ± 2221	18,451 ± 2137	−1328 ± 611	0.64	<0.0001	0.078
IMAT, 20% site	4082 ± 1114	3752 ± 1064	−330 ± 189	3374 ± 1152	3093 ± 1128	−291 ± 219	0.018	<0.0001	0.46
IMAT, 50% site	5386 ± 1528	5004 ± 1573	−282 ± 513	4539 ± 1619	4146 ± 1620	−329 ± 459	0.036	<0.0001	0.90

*Note*: Mean ± standard deviation. Linear mixed models were used to assess the effects of testosterone status group, time, and their interaction on outcome measures. If a significant interaction effect was observed, post hoc comparisons were adjusted for multiple comparisons using the Bonferroni correction. Data not sharing the same lowercase letter within a group are different and *indicates a between group difference at that particular time point (group‐by‐time interaction, *p* < 0.05).

Abbreviations: CSA, cross‐sectional area; IMAT, intramuscular adipose tissue; pQCT, peripheral Quantitative Computed Tomography.

Individuals in the normal testosterone group had a smaller decrease in testosterone compared to those in the low testosterone group in response to SERE (*p*‐interaction = 0.0045; Table 3). Similarly, individuals in the normal testosterone group had a smaller decrease in free testosterone compared to those in the low testosterone group in response to SERE (*p*‐interaction <0.0001; Table 3). Total body mass decreased similarly in both normal testosterone and low testosterone groups (*p*‐time <0.0001); however, FFM decreased less (*p*‐interaction = 0.016) and fat mass more (*p*‐interaction = 0.021), particularly in the trunk region (FFM: *p*‐interaction = 0.044 and fat mass: *p*‐interaction = 0.018), in the normal testosterone versus low testosterone group (Table 2). Leg muscle CSA tended to decrease less in the normal testosterone group compared to the low testosterone group (20% site, *p*‐interaction = 0.068 and 50% site, *p*‐interaction = 0.078; Table 2).

**TABLE 3:** Changes in biochemical measures by post‐SERE hormonal status

	Post‐SERE testosterone status								
	Normal testosterone (*n* = 24, ≥ 10.5 nmol/L)			Low testosterone (*n* = 44, < 10.5 nmol/L)			*p*‐value		
	Pre	Post	Δ	Pre	Post	Δ	Group	Time	Interaction
Testosterone, nmol·L^−1^	19.6 ± 4.5^a*^	14.1 ± 3.4^b*^	−5.5 ± 4.2	16.4 ± 4.4^a^	7.5 ± 1.8^b^	−8.9 ± 4.6	< 0.0001	< 0.0001	0.0045
Free testosterone^a^, pmol·L^−1^	392 ± 92^a^	188 ± 56^b*^	−204 ± 90	359 ± 122^a^	106 ± 32^b^	−254 ± 123	< 0.0001	< 0.0001	< 0.0001
SHBG, nmol·L^−1^	37.9 ± 12.4	64.1 ± 18.4	26.2 ± 10.9	31.2 ± 9.2	54.7 ± 14.0	23.6 ± 9.8	0.012	< 0.0001	0.31
DHEAs^a^, umol·L^−1^	6.73 ± 2.34	9.20 ± 3.51	2.47 ± 2.69	6.58 ± 2.00	8.11 ± 3.02	1.59 ± 2.74	0.39	< 0.0001	0.19
LH^a^, IU·L^−1^	3.94 ± 1.44	4.63 ± 2.20	0.68 ± 1.86	3.85 ± 1.60	5.46 ± 2.36	1.60 ± 2.20	0.54	0.0001	0.067
GH^a^, ug·L^−1^	0.14 ± 0.15^a^	0.90 ± 1.44^b^	0.76 ± 1.41	0.34 ± 0.78^a^	1.00 ± 2.51^b^	0.65 ± 1.99	0.96	< 0.0001	0.047
IGF‐1^a^, nmol·L^−1^	38.4 ± 14.9	24.5 ± 9.3	−13.9 ± 16.9	45.3 ± 18.4	25.8 ± 13.2	−20.1 ± 20.5	0.40	< 0.0001	0.21
Glucose^a^, mmol·L^−1^	4.57 ± 0.19	5.28 ± 0.50	0.71 ± 0.51	4.65 ± 0.31	5.26 ± 0.73	0.61 ± 0.69	0.82	< 0.0001	0.42
Insulin^a^, pmol·L^−1^	25.3 ± 9.7	32.0 ± 20.5	6.7 ± 21.1	24.2 ± 12.0^a^	55.8 ± 56.7^b^	32.1 ± 54.5	0.18	0.0001	0.014
hsCRP^a^, mg·L^−1^	2.02 ± 1.78	3.10 ± 4.21	1.08 ± 4.52	1.59 ± 2.26	2.49 ± 2.96	0.93 ± 3.43	0.28	0.18	0.90
IL6^a^, pg·ml^−1^	8.0 ± 11.0	10.8 ± 13.7	2.9 ± 3.6	6.0 ± 5.2	8.1 ± 7.2	2.2 ± 4.2	0.55	< 0.0001	0.60
NPY, pmol·L^−1^	99 ± 33	67 ± 25	−31 ± 28	100 ± 34	67 ± 27	−33 ± 28	0.95	< 0.0001	0.77
Prolactin^a^, ug·L^−1^	11.1 ± 3.8	7.7 ± 2.8	−3.4 ± 3.2	13.0 ± 5.5	8.4 ± 3.6	−4.6 ± 5.6	0.17	< 0.0001	0.56
Cortisol, nmol·L^−1^	450 ± 94	473 ± 115	23 ± 103	478 ± 115	478 ± 121	2 ± 126	0.53	0.43	0.46
Epinephrine^a^, pmol·L^−1^	302 ± 152	445 ± 201	144 ± 231	317 ± 170	388 ± 185	76 ± 197	0.55	< 0.0001	0.21
Norepinephrine^a^, pmol·L^−1^	3186 ± 1944	3437 ± 1534	250 ± 1757	2605 ± 1318^a^	3902 ± 1498^b^	1343 ± 1727	0.91	< 0.0001	0.016
Amino acids, μmol·L^−1^									
Total	2877 ± 147	3161 ± 405	238 ± 435	2630 ± 593^a^	3450 ± 556^b^	776 ± 759	0.88	0.0001	0.039
Essential	1043 ± 162	1033 ± 182	32 ± 183	918 ± 187^a^	1149 ± 260^b^	232 ± 294	1.00	0.032	0.033
Branched chain	574 ± 108	539 ± 121	13 ± 112	488 ± 104^a^	594 ± 160^b^	121 ± 162	0.80	0.13	0.040
Leucine	162 ± 31	142 ± 37	−12 ± 33	138 ± 27	161 ± 51	24 ± 54	0.91	0.80	0.045

*Note*: Mean ± standard deviation. Linear mixed models were used to assess the effects of testosterone status group, time, and their interaction on outcome measures. If a significant interaction effect was observed, post hoc comparisons were adjusted for multiple comparisons using the Bonferroni correction. Data not sharing the same lowercase letter within a group are different and *indicates a between group difference at that particular time point (group‐by‐time interaction, *p* < 0.05).

^a^ Indicates variable was log transformed for statistical analysis.

Abbreviations: DHEAs, dehydroepiandrosterone sulfate; hsCRP, high‐sensitivity C‐reactive protein; GH, growth hormone; IGF‐1, insulin‐like growth factor 1; IL‐6, interleukin 6; LH, luteinizing hormone; NPY, neuropeptide Y; SHBG, sex hormone‐binding globulin.

SHBG increased from PRE to POST in both groups (*p*‐time <0.0001); however, SHBG was lower in the low testosterone group compared to the normal testosterone group, independent of time (*p*‐testosterone status = 0.012; Table 3). Growth hormone increased from PRE to POST in both the normal and low testosterone groups, but there was a greater change in the normal testosterone group compared to the low testosterone group (*p*‐interaction = 0.047). Insulin concentrations increased from PRE to POST in the low testosterone group (post hoc comparison, *p* < 0.0001), such that change in insulin differed by group (*p*‐interaction = 0.014; Table 3). Norepinephrine concentrations increased from PRE to POST in the low testosterone group (post hoc comparison, *p* < 0.0001), such that change in norepinephrine differed by group (P‐interaction = 0.01; Table 3). Circulating total AA, EAA, and BCAA concentrations increased from PRE to POST in the low testosterone group (post hoc comparison, *p* ≤ 0.05), such that change in total AA, EAA, and BCAA differed by group (*p*‐interaction ≤0.040; Table 3). Change in leucine concentrations also differed by group (*p*‐interaction <0.045; Table 3).

Whole‐body protein synthesis, net balance, and flux decreased and whole‐body protein breakdown increased from PRE to POST in both groups (*p*‐time ≤0.025; Table 4). However, whole‐body protein synthesis, breakdown, and flux were elevated in the low testosterone group compared to the normal testosterone group, independent of time (*p*‐testosterone status ≤0.0036; Table 4).

**TABLE 4:** Changes in whole‐body protein turnover by post‐SERE hormonal status

	Post‐SERE testosterone status								
	Low testosterone (*n* = 24, < 10.5 nmol/L)			Low testosterone (*n* = 44, < 10.5 nmol/L)			*p*‐value		
	Pre	Post	Δ	Pre	Post	Δ	Group	Time	Interaction
Whole‐body protein turnover, g protein·kg^−1^·day^−1^									
Protein synthesis	6.30 ± 0.99	5.63 ± 1.32	−0.68 ± 1.29	7.06 ± 1.27	6.59 ± 1.75	−0.51 ± 2.21	0.0019	0.025	0.69
Protein breakdown	5.08 ± 1.33	6.14 ± 1.46	1.10 ± 1.47	6.09 ± 1.56	7.27 ± 2.06	1.15 ± 2.15	0.0036	< 0.0001	0.85
Net protein balance	1.22 ± 0.89	−0.51 ± 0.31	−1.78 ± 0.84	0.96 ± 1.30	−0.68 ± 0.45	−1.66 ± 1.21	0.23	< 0.0001	0.74
Flux	1.21 ± 0.20	0.98 ± 0.23	−0.23 ± 0.22	1.36 ± 0.19	1.16 ± 0.33	−0.20 ± 0.36	0.0020	< 0.0001	0.66

*Note*: Mean ± standard deviation. Linear mixed models were used to assess the effects of testosterone status group, time, and their interaction on outcome measures. If a significant interaction effect was observed, post hoc comparisons were adjusted for multiple comparisons using the Bonferroni correction.

## 4 DISCUSSION

This secondary analysis evaluated metabolic and physiological differences between US Marines with low testosterone (defined as below the 5th percentile for normal total testosterone concentrations in healthy, nonobese males 19–39 y, < 10.5 nmol/L) compared to those with normal testosterone (≥ 10.5 nmol/L) following 7 days of severe energy deficit and stress resulting from SERE training. This study showed that participants who maintained normal testosterone concentrations following training, compared to those who experienced low testosterone concentrations had, (1) greater BMI, total fat mass, trunk fat mass, and testosterone concentrations and a lower diet quality score and total carbohydrate intake at baseline, (2) less fat‐free mass loss and more fat mass loss, particularly in the trunk region, in response to training, (3) different circulating total testosterone, free testosterone, growth hormone, norepinephrine, insulin, and amino acid responses to training, and (4) lower overall rates of whole‐body protein synthesis, breakdown, and flux.

In the current study, participants who maintained normal testosterone concentrations following 7 days of severe energy deficit as a result of SERE training had greater BMI, total fat mass, trunk fat mass, and testosterone concentrations prior to the start of training than participants who experienced low testosterone concentrations following the same military training. Greater testosterone concentrations prior to training may have allowed reductions in testosterone to occur in response to the severe energy deficit without concentrations becoming low (i.e., < 10.5 nmol/L). However, this does not fully explain the preservation of testosterone concentrations in the normal testosterone group since this group also had smaller decrements in testosterone following training compared to the low testosterone group. Furthermore, lower diet quality, including greater solid fat, alcohol, and added sugar (SoFAAS) intake, and less total carbohydrate intake before training were dietary habits in the group that maintained normal testosterone concentrations. This lower diet quality may have contributed to greater total and trunk fat mass observed in the normal testosterone group prior to training.

Testosterone and body mass decrements have been observed during both moderate and severe energy deficits in healthy normal weight males, although the amount of fat‐free and fat mass loss varies widely (Alemany et al., 2008; Henning, Margolis, et al., 2014; Henning, Scofield, et al., 2014; Kyröläinen et al., 2008; Nindl, Alemany, et al., 2007; Nindl, Barnes, et al., 2007; Øfsteng et al., 2020; Pasiakos et al., 2013). In the current study, participants who maintained normal testosterone concentrations following a 7‐day severe energy deficit lost less whole‐body and trunk fat‐free mass and more whole‐body and trunk fat mass compared to those who experienced low testosterone concentrations following training. However, both groups lost similar amounts of appendicular fat‐free mass and fat mass and IMAT in the thigh following training, suggesting the relationship between testosterone concentrations and body composition during energy deficit may differ by body depot. Greater fat mass at the onset of training may have served, in part, as a protective factor, as more pre‐training fat mass was associated with greater decrements in fat mass (*r* = −0.35, *p* = 0.0031) and smaller declines in testosterone (*r* = 0.57, *p* < 0.0001). Consistent with Forbes' Theory, initial fat mass was inversely associated with fat‐free mass loss as a proportion of total body mass loss (i.e., ∆ fat‐free mass/∆ total body mass, *r* = −0.25, *p* = 0.042). Forbes' Theory suggests that individuals with less initial fat mass lose more fat‐free mass as a proportion of total body mass loss during periods of negative energy balance (Forbes, 1987, 2000). A study by Friedl et al. (1994) investigated body composition changes in healthy males following an 8‐week military training course that involved severe energy deficit and stress. Based on the study findings, the authors suggest there may be a lower limit of body fat (4%–6% or 2.5 kg) and, once reached, any further mass loss will come from the fat‐free compartment, making greater initial fat mass protective during periods of severe energy deficit and training. Identifying preemptive physical traits, nutritional habits, and exercise regimens associated with attenuated testosterone decrements during periods of unavoidable energy deficit may lead to better preparedness in military personnel and facilitate improved implementation of military training.

Fasting and energy deficiency are known to reduce testosterone concentrations in males, which has been suggested as an adaptive response to minimize energy requirements (De Souza et al., 2019; Hackney, 2020; Lane & Hackney, 2014; Mountjoy et al., 2018). In the current study, due to the greater loss of absolute fat mass in the normal testosterone group, the energy deficit determined from changes in energy stores (i.e., fat mass and non‐aqueous fraction of fat‐free mass) was greater in the normal testosterone group (−4761 ± 1893 kcal/d) compared to the low testosterone group (−3845 ± 1433 kcal/d, *p* = 0.028). Since both groups had a similar estimated basal metabolic rate prior to training (~1800 kcal/d), performed the same physical tasks, and were provided the same amount of food, the smaller energy deficit in the low testosterone group may indicate energy requirements were attenuated in this group during training. Although the normal testosterone group lost more absolute fat mass in response to training, they still had a greater percentage of fat mass at PRE‐ and POST‐SERE compared to the low testosterone group (*p*‐group = 0.008). These findings may be explained, in part, by leptin (not measured in the current study), an adipokine secreted in proportion to body fat, which indirectly signals for the secretion of gonadotrophin releasing hormone (GnRH) via kisspeptin within the hypothalamus (Navarro, 2020). Gonadotrophin releasing hormone (not measured in the current study) acts on the pituitary to signal the release of LH (signals for testosterone production by the testes), GH (involved in anabolic processes, signals for IGF‐1 production by the liver), and prolactin (upregulates LH receptors). Less fat mass likely results in less leptin production, which may be influencing testosterone concentrations in the current study. This is supported by findings that greater POST fat mass was correlated with greater POST testosterone concentrations (*r* = 0.28, *p* = 0.020) and a smaller decrease in testosterone concentrations (*r* = 0.51, *p* < 0.0001).

A previous study investigating the effects of short‐term (84 h) military‐relevant stress (i.e., energy restriction, physical exertion, and sleep deprivation) on endocrine function reported decreased leptin, testosterone, and IGF‐1 concentrations and increased LH and GH concentrations during military‐relevant stress compared to the ad libitum control condition (Nindl et al., 2006). The authors suggest that an increase in LH and a concomitant decrease in testosterone indicates peripheral resistance to LH; similarly, the authors suggest an increase in GH with a concurrent decrease in IGF‐1 may indicate decreased GH sensitivity in the liver. In the current study, both groups experienced an increase in LH and decrease in testosterone concentrations, but the low testosterone group had a greater decrease in testosterone concentrations with a similar increase in LH to the normal testosterone group. However, without having measured LH pulse characteristics, it is not possible to determine whether this difference is due to greater peripheral resistance to LH, gonadal inadequacy, or dysregulation by other factors such as stress hormones in the low testosterone group. Furthermore, in the current study, GH concentrations increased in both groups but to a greater extent in the normal testosterone group, with both groups experiencing similar decrements in IGF‐1. The greater increase in GH concentrations following SERE may be independent of the effects of GnRH, with other factors (e.g., magnitude of energy deficit, norepinephrine concentrations) potentially contributing to the difference between groups. Interestingly, GH stimulates lipolysis and fat mobilization (Carrel & Allen, 2000), which occurred to a greater extent in the normal testosterone group (i.e., greater fat mass loss).

In the current study, epinephrine was increased in both groups, norepinephrine was increased in the low testosterone group, and cortisol did not change in response to SERE training. The normal testosterone group had PRE‐SERE norepinephrine values above the normal range (1270–2810 pmol/L) that did not increase in response to training, whereas the low testosterone group had normal norepinephrine concentrations at PRE‐SERE that increased in response to training. This difference in responses may have been due to a greater perception of stress in the low testosterone group leading to more norepinephrine production or greater conversion of norepinephrine to epinephrine in the normal testosterone group. Previous research has shown differences in norepinephrine responses to strenuous training and recovery depending on fitness level and special forces (SF) status (Morgan et al., 2001; Szivak et al., 2018). Specifically, increased norepinephrine responses to a 12‐h simulated interrogation in SF Soldiers compared to non‐SF Soldiers (Morgan et al., 2001) and lower norepinephrine concentrations in high fit compared to low fit participants following 24‐h of recovery from SERE training (Szivak et al., 2018). Surprisingly, despite the increase in norepinephrine in the low testosterone group, cortisol did not change in either group POST‐SERE. This finding is in agreement with one previous study (Opstad, 1992) and at odds with several other survival‐training studies, which report a decrease (Lieberman et al., 2016) or large increase (Morgan et al., 2000; Szivak et al., 2018) in cortisol following survival training. Similar to the study by Opstad (1992), in the current study, both groups had cortisol concentrations above the normal range (50–410 nmol/L) before and after survival training. This suggests participants started training in a more stressed state than in some previous studies, which may indicate a heavy training load prior to SERE school and help explain the low‐normal testosterone concentrations at PRE (17.5 ± 4.7 nmol/L).

Reductions in circulating testosterone concentrations in response to food deprivation and strenuous physical training may be due to less testosterone production, as discussed above, and/or greater uptake of testosterone by body tissues, particularly skeletal muscle. Physical and mental stress, both before and during training in the current study, may have led to greater cellular uptake of testosterone by skeletal muscle androgen receptors to signal protein synthesis (via genomic and non‐genomic androgen receptor signaling) and inhibit catabolism (via interference with the glucocorticoid receptor and cortisol binding) (Kraemer et al., 2020). The normal testosterone group was able to maintain greater fat‐free mass despite a smaller decrease in circulating endogenous testosterone concentrations, which may indicate less androgen receptor binding but more efficient utilization of testosterone by the muscle. Future studies should include measures of cellular testosterone concentrations, androgen receptor number and sensitivity, and blood flow to better characterize molecular and functional factors that may be influencing circulating testosterone concentrations.

Interestingly, there were greater overall rates of whole‐body protein synthesis, breakdown, and flux in the low testosterone group compared to the normal testosterone group, independent of time. During PRE‐SERE, the trend for greater protein intake and significantly higher whole‐body protein flux values in the low testosterone group was consistent with the need for greater nutritional signals (i.e., protein and EAA) to maintain a similar muscle mass to those with greater anabolic signaling by endogenous testosterone concentrations (i.e., the normal testosterone group). This is also consistent with findings that testosterone improves the reutilization of intracellular AA (Ferrando et al., 1998), thereby reducing the turnover ratio of protein synthesis to protein breakdown.

The POST‐SERE increase in glucose and decrease in NPY concentrations in both groups and increase in insulin concentrations in the low testosterone group is hard to reconcile with previous research on strenuous training involving periods of energy deficit and stress. Previous studies have typically reported no change or reductions in glucose and insulin (Chan et al., 2003; Friedl et al., 2000; Nindl, Alemany, et al., 2007; Nindl et al., 2006) and no change or increases in NPY (Lieberman et al., 2016; Szivak et al., 2018) immediately following training. In the current study, the increase in glucose and insulin and decrease in NPY may be due to the timing of the blood draws. Participants completed training between 1700–1800 h and DEXA measurements were collected immediately. Then, participants were able to consume food, but asked to fast for 8–10 h prior to the blood draw the following morning. Despite being fasted for the morning blood draw, allowance of the food immediately POST may have altered circulating glucose, insulin, NPY, and other biomarker concentrations and may limit comparability of POST‐SERE measures in the current study with previous studies of strenuous military training.

A limitation of the current analysis is the lack of physical performance measures to determine whether those who maintained normal testosterone concentrations performed better during SERE training than those with low testosterone. We have previously shown that exogenous testosterone administration during a 55% energy deficit does not improve functional measures when compared to placebo (Pasiakos et al., 2019). Based on this previous study, testosterone supplementation would not be recommended to restore eugonadal concentrations during strenuous, short‐term military training in healthy male military personnel. However, future studies should evaluate testosterone supplementation within this context on military‐relevant physical performance and long‐term health. The study of testosterone restoration during severe energy deficit to improve performance and health in highly specialized military personnel should not be confused with illicit use of anabolic‐androgenic steroids at supraphysiological doses to improve performance in athletes or recreational users. Recent increases (20%–30%) in androgen prescriptions for civilian and military populations (Canup et al., 2015) underscore the need for accurate clinical diagnosis of hypogonadism and careful consideration of the risks and benefits of treatment before initiating androgen therapy (Bhasin et al., 2021).

Other limitations include the single measurement of testosterone at both PRE‐ and POST‐SERE using an immunoassay (Siemmens Immulite 2000), which is less accurate than LCMS (La'ulu et al., 2018). Total testosterone concentrations are lower when measured by Immulite compared to LCMS; in a previous study, when samples were measured by Immulite, 21% of individuals had testosterone concentrations <11 nmol/L compared to 8% with LCMS (Montagna et al., 2018). This may help explain the low‐normal testosterone concentrations at PRE and could have led to classification of more individuals as having low testosterone at POST than if the gold‐standard LCMS had been used to measure testosterone concentrations. Furthermore, to diagnose hypogonadism, the Endocrine Society Clinical Practice Guideline recommends conducting a medical history and physical exam, measuring fasting morning testosterone concentrations, and then confirming a low testosterone outcome by repeat measurement (Bhasin et al., 2018). In the current study, all participants were subjected to a uniform stressor that can cause secondary hypogonadism (i.e., nutritional deficiency/excessive exercise) and their testosterone was measured in the morning after an overnight fast. A repeat measure of testosterone at both PRE and POST would have increased data reliability; however, study findings are strengthened by the consistent timing of fasted morning blood samples, relatively homogenous population, and standardized stressor.

Furthermore, resistance exercise acutely increases total and free testosterone concentrations and muscle androgen receptor mRNA and protein content (Kraemer et al., 2020), whereas eating immediately following a bout of resistance exercise reduces total testosterone concentrations and increases muscle androgen receptor content for the subsequent hour (Kraemer et al., 2006). Therefore, it is a limitation that exercise was not restricted 48 h prior to collecting the PRE‐SERE blood sample; however, the 8–10 h fast prior to both the PRE and POST‐SERE blood samples and the uniform physical load prior to POST‐SERE measures likely minimize the variability due to nutrition, exercise, and sleep. In addition, energy deficient diets are known to increase SHBG (Cangemi et al., 2010; Chan et al., 2003; Friedl et al., 2000; Henning, Scofield, et al., 2014) and the increase in SHBG at POST may decrease the validity of the free testosterone calculation. Finally, the use of predictive equations (i.e., BMR, change in energy stores, TDEE) to calculate variables may have been a source of error. Strengths of the current study include the uniform stressor applied during SERE training, the large sample size, and the comprehensive panel of metabolic and endocrine markers that were measured.

In conclusion, low testosterone (below the 5th percentile for normal total testosterone concentrations in healthy, nonobese males 19–39 y, < 10.5 nmol/L) following SERE training is associated with greater fat‐free mass loss and less fat mass loss in response to this training. Altering body composition and dietary strategies prior to SERE training may provide an opportunity to attenuate post‐training decrements in testosterone and minimize the detrimental/catabolic consequences of stressful military training and operations. Furthermore, maintenance of normal testosterone during training may be indicative of a more resilient phenotype, which may identify individuals who are better suited for subsequent high‐stress/specialized training and may need less recovery time between consecutive missions. Interventions that prevent decrements in testosterone during strenuous military training should be investigated as a method to reduce fat‐free mass loss, potential physical performance declines, and other detrimental consequences of low testosterone.

## AUTHOR CONTRIBUTIONS

H.L.M, A.A.F, E.G.‐S., J.P.M, and S.M.P. were responsible for conception and design of research; H.L.M., J.J.S., E.G.‐S., J.P.M, and S.M.P. performed experiments; C.E.B. and S.M.P. analyzed data; C.E.B. wrote the manuscript; all authors edited and revised the manuscript and approved the final version of the manuscript.

## FUNDING INFORMATION

Supported in part by the U.S. Army Medical Research and Development Command and appointment to the U.S. Army Research Institute of Environmental Medicine administered by the Oak Ridge Institute for Science and Education through an interagency agreement between the U.S. Department of Energy and the U.S. Army Medical Research and Development Command.

## CONFLICT OF INTEREST

The authors have nothing to disclose. The opinions or assertions contained herein are the private views of the authors and are not to be construed as official or reflecting the views of the Army or Department of Defense. Any citations of commercial organizations or trade names in this report do not constitute official Department of the Army endorsement or approval of the products or services of these organizations.

## Supporting information


Table S1
