# Supplementary material for: Testosterone status following short‐term, severe energy deficit is associated with fat‐free mass loss in U.S. Marines
Source: Physiol Rep. 2022 Sep 18;10(18):e15461. doi: 10.14814/phy2.15461 (PMC9483439; doi:10.14814/phy2.15461)
Supplement: Supplementary file 1 — Table S1 [file PHY2-10-e15461-s001.docx]

**Supplemental Table 1.** Correlations between POST testosterone concentrations and PRE to POST SERE change in body composition, hormones, inflammatory markers, catecholamines, and protein synthesis and breakdown measures.

|  | Post TT | ∆TT^1^ | ∆FFM | ∆FM | ∆SHBG | ∆IGF1 | ∆LH | ∆GH^1^ | ∆Glu^1^ | ∆Ins^1^ | ∆CRP^1^ | ∆IL6 | ∆Epi^1^ | ∆Nor^1^ | ∆Cort | ∆PS^1^ | ∆PB |
| --- | --- | --- | --- | --- | --- | --- | --- | --- | --- | --- | --- | --- | --- | --- | --- | --- | --- |
| Post TT | 1.00 |  |  |  |  |  |  |  |  |  |  |  |  |  |  |  |  |
| ∆TT^1^ | 0.70*** | 1.00 |  |  |  |  |  |  |  |  |  |  |  |  |  |  |  |
| ∆FFM | 0.37** | 0.26* | 1.00 |  |  |  |  |  |  |  |  |  |  |  |  |  |  |
| ∆FM | -0.26* | -0.19 | -0.60*** | 1.00 |  |  |  |  |  |  |  |  |  |  |  |  |  |
| ∆SHBG | 0.14 | 0.10 | 0.00 | 0.01 | 1.00 |  |  |  |  |  |  |  |  |  |  |  |  |
| ∆IGF1 | 0.11 | 0.02 | 0.09 | -0.13 | -0.08 | 1.00 |  |  |  |  |  |  |  |  |  |  |  |
| ∆LH | -0.17 | 0.03 | -0.08 | 0.01 | -0.07 | -0.01 | 1.00 |  |  |  |  |  |  |  |  |  |  |
| ∆GH^1^ | 0.31* | 0.39** | 0.18 | -0.10 | 0.01 | 0.06 | -0.22 | 1.00 |  |  |  |  |  |  |  |  |  |
| ∆Glu^1^ | 0.05 | -0.07 | 0.02 | 0.06 | -0.10 | 0.04 | -0.15 | -0.07 | 1.00 |  |  |  |  |  |  |  |  |
| ∆Ins^1^ | -0.39** | -0.44*** | -0.22 | 0.17 | -0.23 | -0.21 | 0.31* | -0.57*** | 0.26* | 1.00 |  |  |  |  |  |  |  |
| ∆CRP^1^ | -0.02 | 0.08 | 0.19 | -0.06 | -0.06 | 0.00 | 0.07 | -0.11 | 0.09 | 0.10 | 1.00 |  |  |  |  |  |  |
| ∆IL6 | 0.03 | -0.19 | 0.08 | 0.04 | -0.06 | 0.20 | -0.30* | 0.01 | 0.26* | 0.05 | 0.26* | 1.00 |  |  |  |  |  |
| ∆Epi^1^ | 0.08 | 0.00 | 0.05 | -0.06 | 0.25* | -0.06 | -0.21 | 0.24 | -0.02 | -0.14 | 0.10 | 0.13 | 1.00 |  |  |  |  |
| ∆Nor^1^ | -0.21 | -0.09 | -0.03 | 0.05 | -0.05 | -0.11 | -0.01 | 0.15 | -0.02 | 0.09 | 0.09 | 0.08 | 0.15 | 1.00 |  |  |  |
| ∆Cort | 0.04 | -0.05 | 0.15 | -0.21 | 0.07 | -0.13 | -0.31* | -0.06 | 0.07 | -0.04 | 0.12 | 0.13 | 0.30* | -0.01 | 1.00 |  |  |
| ∆PS^1^ | -0.11 | -0.28* | -0.03 | -0.04 | 0.09 | -0.05 | -0.32** | -0.20 | 0.17 | 0.02 | 0.09 | 0.19 | 0.19 | 0.05 | 0.25* | 1.00 |  |
| ∆PB | -0.10 | -0.26* | -0.05 | 0.09 | 0.15 | -0.06 | -0.20 | -0.32* | 0.11 | 0.01 | 0.04 | 0.17 | 0.16 | -0.01 | 0.15 | 0.83*** | 1.00 |
| ∆EAA | -0.49** | -0.26 | -0.28 | 0.14 | -0.18 | -0.09 | -0.04 | -0.20 | 0.20 | 0.37 | -0.43* | 0.12 | 0.02 | 0.15 | 0.28 | -0.31 | -0.32 |

Data represents Pearson’s or Spearman’s correlation coefficients. Spearman’s correlation was used for any variable that did not demonstrate a normal distribution before or after transformation (∆FM and ∆IL6). Pearson’s correlation was used when change scores for both variables (before or after a log10-transformation) demonstrated a normal distribution. *P < 0.05, **P < 0.01, ***P < 0.001. Abbreviations: Cort, Cortisol; CRP, high-sensitivity C-reactive protein; EAA, essential amino acids; Epi, epinephrine; FFM, fat-free mass; FM, fat mass; GH, growth hormone; Glu, glucose; IGF-1, insulin-like growth factor 1; IL-6, interleukin 6; Ins, insulin; LH, luteinizing hormone; Nor, norepinephrine; PB, protein breakdown; POST, assessments obtained immediately after SERE training; PRE, assessments obtained before SERE training; PS, protein synthesis; SERE, Survival, Evasion, Resistance, Escape training; SHBG, sex hormone-binding globulin; TT, total testosterone.

^1^Indicates variable was log transformed for statistical analysis.
